# Effects of Interprofessional Education on Readiness for Interprofessional Learning in Rehabilitation Science Students From Professional Health Care Programs: Protocol for a Systematic Review

**DOI:** 10.2196/60830

**Published:** 2024-11-20

**Authors:** Eric Dixon, Jayden Pannu, Kabir Dhaliwal, Rachel Cheng, Gurpal Deol, Sophie Frangos, Emma Tawil, Ana Oliveira, Sarah Wojkowski, Shirley Quach

**Affiliations:** 1 School of Rehabilitation Sciences Faculty of Health Sciences McMaster University Hamilton, ON Canada; 2 Lab3R – Respiratory Research and Rehabilitation Laboratory School of Health Sciences University of Aveiro (ESSUA) Aveiro Portugal; 3 Program for Interprofessional Practice, Education and Research (PIPER) Faculty of Health Sciences McMaster University Hamilton, ON Canada

**Keywords:** interprofessional education, rehabilitation science, health care students, interprofessional collaboration, prelicensure health care professionals, patient care, interdisciplinary education, rehabilitation education, curriculum development, team-based learning

## Abstract

**Background:**

The World Health Organization defines interprofessional education (IPE) as a process in which students from different health care programs work together to provide effective care while deepening their knowledge of each other’s roles. Previous literature shows a strong argument for early exposure to IPE as a facilitator for high quality patient care. The goal of IPE is to improve interprofessional collaboration (IPC), the “gold standard” of care to enhance patients' quality of life, functional ability, and health status, especially for patients who require expertise from a variety of health care professionals. IPC has shown improvements in quality of life, functional ability, and health status. IPE can occur in the form of structured interventions or spontaneously in student placements. Literature has demonstrated that IPE facilitates skill, knowledge development, teamwork, communication skills, and mutual respect among health care professional students.

**Objective:**

This systematic review aims to examine IPE outcomes, including readiness for IPC, IPE perceptions, attitudes toward collaborative learning, student confidence, practice efficiency, and team dynamics after IPE interventions in rehabilitation science students.

**Methods:**

The study will be conducted as outlined by the Cochrane Handbook for Systematic Reviews and will be reported per the PRISMA (Preferred Reporting of Items for Systematic Reviews and Meta-Analyses) 2015 guidelines. Students have performed literature searches across the databases MEDLINE, Embase, CINAHL, ERIC, Web of Science, and AMED. Studies will be included if their IPE intervention included multiple prelicensure health care professional students in a health care or health care education setting. Based on timelines presented in the Institute of Medicine’s report on the impacts of IPE, relevant studies from 2016 to the present will be included. The Risk of Bias 2 tool will be used to study sources of bias. The GRADE (Grading of Recommendations, Assessment, Development, and Evaluation) working group’s methods will be used to evaluate the quality of the evidence presented. The final 3 authors are assisting as supervisors, providing oversight and feedback as needed. Any deviations from this protocol will be reported in the final paper.

**Results:**

The search strategy was finalized and searched across the databases by March 8, 2024. The systematic review was registered with PROSPERO on March 31, 2024. A total of 10,692 citations were retrieved for abstract and title screening, beginning in March 2024, and 756 were eligible for full-text screening in April 2024. Six articles were considered for inclusion and data extraction, which began in July 2024. Finalization of the extracted data and paper will occur in September 2024.

**Conclusions:**

This systematic review will provide a summary of the effects of IPE interventions in prelicensure rehabilitation science students. It will provide educators, health care providers, and students with valuable information for understanding the relevance of IPE. It will also shed light on research gaps and highlight areas for further study.

**Trial Registration:**

PROSPERO CRD42024506081; https://tinyurl.com/3tf2h9er

**International Registered Report Identifier (IRRID):**

PRR1-10.2196/60830

## Introduction

Chronic diseases currently affect 1 in 3 Canadians and the World Health Organization (WHO) predicts that this will result in 52 million deaths by 2030 [1]. The high multisystemic impact of chronic diseases on patients and health care systems underscores the critical need for top-tier health care [1,2]. Top-tier health care can be facilitated with the early integration of interprofessional education (IPE) in health care students’ curricula [3].

Various definitions of IPE exist in the literature. A widely accepted, global definition from the WHO defines IPE as the collaboration among students from different health care professionals to deepen their understanding of each other’s roles and who work together to provide safe and effective care [4]. This form of education can occur in structured activities integrated into the curriculum or arise spontaneously during student placements [4]. IPE aims to enhance clinical practice by facilitating knowledge and skills development in teamwork, communication skills, and mutual respect among health care professional students [5-8]. Therefore, IPE should be considered pivotal in providing comprehensive care.

In educational settings, systematic reviews have found that students and licensed health care professionals participate in IPE in several ways [9-11]. This includes small group discussions, simulations, and workshops, which led to improved attitudes and perceptions of IPE, confidence, and self-efficacy associated with team-based performance and communication skills [9,11]. Notably, over half the studies in both reviews showed significant improvements in positive attitudes, suggesting the effectiveness of these interventions [9,11]. While treatments have been proven effective, a review looking at a follow-up period reported that improvements were only sustained for a short period [9]. Moreover, the same review saw improvements in patient outcomes such as patient safety, time from arrival to care, and pressure ulcers, but no differences in length of stay, mortality rate, and complications in the hospital setting with the implementation of IPE [9]. Although many positive improvements were noted, these reviews reported very low quality of evidence and heterogeneity across studies, due to the lack of randomized controlled trials (RCTs), a high proportion of loss to follow-up, and a high risk of bias (RoB) [9,11]. These IPE effects were predominantly reported in nursing and medical students [9,11], with very few studies focusing on other health care professional students, such as those enrolled in rehabilitation programs.

Rehabilitation consists of interventions aimed at optimizing the quality of life in individuals with health conditions [12]. Along with increasing independence and promoting participation in meaningful activity, rehabilitation contributes to healthy aging through disease management and helps reduce morbidity and mortality [12]. According to WHO, rehabilitation professionals include “physiotherapists, occupational therapists, speech and language therapists and audiologists, orthotists and prosthetists, clinical psychologists, physical medicine and rehabilitation doctors, and rehabilitation nurses” [12]. Exploring the effects of IPE on rehabilitation science students is crucial, as it can contribute to improved chronic disease management and enhanced quality of health care delivery through collaboration among various professionals.

Thus, the goal of this systematic review is to explore the effects of IPE on prelicensure rehabilitation science students’ readiness toward interprofessional collaboration (IPC). Secondary outcomes will also be explored, including but not limited to team dynamics, confidence levels, closed-loop communication, and practice efficiency.

## Methods

### Overview

This systematic review will be conducted as outlined by the Cochrane Handbook of Systematic Reviews and reported as per the updated PRISMA (Preferred Reporting Items for Systematic Reviews and Meta-Analysis) 2015 statement [13-15]. The protocol has been registered in PROSPERO (CRD42024506081) and the PRISMA Protocols (PRISMA-P) checklist was used to guide this protocol submission [15]. If any deviations occur from this protocol, this will be reported in the final manuscript.

### Eligibility Criteria

Studies will be selected according to the population, intervention, comparison, and outcome (PICO) framework and will be limited to those conducted between 2000 and 2024 to reflect contemporary educational practices and the prominence of IPE in rehabilitation professions' curricula. This was aligned with the rise in implementation of IPE initiatives in the 2000s across health care systems—such as in Canada where IPE and collaboration began to gain traction and were reported to be “essential to achieving effective delivery of health care” [16]. Furthermore, prelicensure rehabilitation training program curricula underwent stark changes in the 2000s, as seen with the World Confederation for Physical Therapy developing new guidelines for entry-level physical therapy education between 2003 and 2011 [16,17].

For title and abstract screening, inclusion criteria were defined by whether the abstract and title addressed the following three questions: (1) “Was there an IPE intervention performed?” (2) “Were multiple health care professions' prelicensure students represented in the study?” (3) “Did the study take place in a health care or health care education setting?” Studies that did not answer “yes” to all 3 statements were excluded. These 3 qualifying questions were derived from the PICO framework to facilitate the screening process. Similarly, in full-text screening, all 3 questions must be satisfied (eg, “yes”) before the studies could be included in this review (Multimedia Appendices 1 and 2).

### Participants

Studies evaluating prelicensure students in entry-to-practice rehabilitation training programs will be included. Rehabilitation disciplines will be defined according to the WHO, including “physiotherapists, occupational therapists, speech and language therapists and audiologists, orthotists and prosthetists, clinical psychologists, physical medicine and rehabilitation doctors, and rehabilitation nurses” [12]. Studies with participants including only practicing rehabilitation professionals will be excluded. For example, registered physiotherapists involved in an IPE opportunity for their professional development requirements.

### Intervention

IPE interventions will be included if presenting two or more rehabilitation science students engaging in an active exchange of information and participation in activities aimed at improving communication and collaboration skills, as per the WHO and the Cuff 2013 definitions [4,18]. Interventions may take place within various settings including large group classes, small group tutorials, clinical settings, and digital platforms [3]. Models of learning may include team-based learning, simulation, and student-led interprofessional clinics [3]. Additional IPE settings and modes of learning not mentioned above may still be included, as long as they satisfy the IPE intervention criteria mentioned above [4,18].

### Comparison

Having a control or comparator group is not a requirement for inclusion in this review. If the study has a control group, the intervention may include usual teaching, learning practice, or any other type of intervention, excluding IPE.

#### Outcome

The primary outcome of this review will be IPE interventions' effect on rehabilitation science students’ readiness for IPC, defined by their interest and ability to work with other professional teams [7,18,19]. This primary outcome was also an important learning outcome outlined in the Interprofessional Learning Continuum model published by the Institute of Medicine [7]. Readiness for IPC may be captured using various measurement tools, which may include but are not limited to, the Readiness for Interprofessional Learning Scale [20], the Student Perceptions of Interprofessional Clinical Education-Revised, the Interdisciplinary Education Perception Scale, the Entry Level Interprofessional Questionnaire [21], and Brief Attitudes Survey for Interprofessional Collaborative Learning [22]. There are numerous outcome measures to assess IPE competencies, but for the purposes of this review, the foci will be tools that evaluate students’ attitudes and abilities for IPC and teamwork [19]. Secondary outcomes that will be examined include students’ perception of IPC, students’ appreciation for the role and scope of complimentary rehabilitation disciplines, closed-loop communication, confidence levels, practice efficiency, and team dynamics. The secondary outcomes may be measured by tools such as the Anesthetists' Non-Technical Skills checklist and purpose-built questionnaires [23]. Outcome measures used to assess primary and secondary outcomes will not be limited in this review.

#### Study Design

This systematic review will include randomized and non-RCTs, cohort studies, and case-control studies published in peer-reviewed journals. Narrative reviews, scoping reviews, and systematic reviews will be excluded; however, their references will be searched for relevant articles. Other documents that will be excluded from this systematic review include abstracts, dissertations, theses, editorials, conference proceedings, magazines, news, and any other non–peer-reviewed papers (Multimedia Appendix 3).

### Search Strategy

A search strategy was developed in consultation with a health sciences librarian at McMaster University to ensure it captured the PICO elements of the research question. To develop this search strategy, the authors identified a set of MeSH (Medical Subject Headings) terms and keywords by examining previous systematic reviews for topics related to IPE, rehabilitation health care personnel, and learning, to find terms linked to these 3 concepts [10,24]; these were then verified with the health sciences librarian. The combination of terms, including the necessary vocabulary and targeted MeSH terms, were trialed in MEDLINE and was similar to: (“health science learners” OR “health science students”) AND (“interprofessional education” OR “interprofessional learning”). The full search string for MEDLINE is available in Multimedia Appendix 4. MEDLINE was chosen as the database to trial the initial search as it is widely considered to be the gold standard for health-related literature searches [25]. Additionally, MEDLINE is most often used as a starting point in systematic review searching due to its extensive collection of biomedical and health-related literature [13,25].

Following the initial search on MEDLINE in February 2024, the search was translated to 4 additional databases including Embase, CINAHL, ERIC, and AMED. These additional databases were selected as they contain education and rehabilitation profession-specific literature relevant to this review. The databases were searched between January 1, 2000, and March 8, 2024, to include IPE interventions and studies after the implemented changes in prelicensure rehabilitation training program curricula in the 2000s [17].

### Study Selection

Literature search results were downloaded from the databases and imported into EndNote 21 (Clarivate) for a first line of duplication removal. The remaining citations were uploaded into Covidence (Veritas Health Innovation) for further duplication removal, followed by title or abstract, and full-text screening. Covidence is a web-based collaboration software platform that streamlines the production of systematic and other literature reviews.

Before engaging in the formal screening process, title and abstract screening were pilot-tested by all authors by reviewing the first 30 texts as a group, ensuring agreement and consistency between all reviewers. According to the recently published best practice guidelines for conducting systematic reviews, it is recommended that abstract screening take place by screening 20 to 30 abstracts to ensure the review team reaches consensus [26]. Three pairs of reviewers (ED, GD, JP, KD, RC, SF, and ET) screened the remainder of the titles and abstracts for inclusion criteria (Multimedia Appendix 1). Each study required consensus between 2 blinded reviewers before a final decision to include or exclude was made. Disagreement resolution was first attempted by discussion, followed by a decision by a third reviewer (SQ, SW, and AO) if consensus cannot be achieved. Following the completion of the title and abstract screening, the full-text screening process included pilot testing, independent reviews, and disagreement resolution using the same framework as the title and abstract screening (Multimedia Appendix 2).

### Data Extraction

Data extraction was pilot-tested by all reviewers by reading the first 10 studies as a group and inputting relevant information into the data extraction table. The data extraction table was reviewed such that all authors are in consensus for the data extraction of the remaining studies. This clarified discrepancies and ensured adequate interrater reliability with data extraction [13,27]. Ten studies were piloted for extraction to ensure that relevant information for data analysis and synthesis was captured. After pilot testing was completed, 3 groups of 2 authors (ED, GD, KD, SF, ET, and RC) were involved in data extraction for all full texts included in the review. The 2 reviewers independently assessed each paper into a predefined data extraction table as outlined in Multimedia Appendix 5. Disagreements were resolved first by discussion, followed by a decision by a third reviewer (SQ, SW, and AO) if consensus could not be achieved.

The chosen information to be extracted aligns with the Cochrane Handbook of Systematic Reviews [13]. Once data extraction was piloted, the tool was modified to ensure all relevant data were captured. The following information will be extracted from studies that met the inclusion criteria: title, author, year, country of publication, study design, and sample size (total and per group). Participant characteristics to be extracted include age (mean and SD), gender, previous IPE experience, previous degrees conferred, and educational programs. Intervention characteristics to be extracted are the level of study, setting, frequency, and duration for both experimental and control groups as well as the timing of data collection. For all of the primary and secondary outcomes, the following data will be extracted: total and subscale scores of the included outcome measures, baseline score and postintervention scores (mean and SD) in both the experimental and control groups, mean group difference and SD, group *P* value, effect size, and a summary of the results. If any of the earlier information is missing from the included studies, authors will be contacted to request missing data. If the data are reported only in figures and the authors do not provide the requested data, WebPlotDigitizer will be used if possible.

### RoB Assessment

The Cochrane RoB tool will be used (randomization process, deviations from intended interventions, missing outcome data, measurement of the outcome, and selection of the reported result) for RCTs within this review [28]. For non-RCTs, the RoB in Non-Randomized Studies of Interventions will be used [29]. Two authors will independently perform the RoB assessment for each citation, any disagreements will be resolved through discussion. If no consensus can be established, a third reviewer will be consulted (SQ, SW, and AO). For this review, RoB will be performed at a study level.

### Confidence in Included Results

Prior to assessing the quality of evidence for each outcome, each study will be assessed for inclusion using the GRADE (Grading of Recommendations Assessment, Development and Evaluation) framework for quality assessment [30]. The quality of evidence will be assessed across the domains of RoB, consistency, directness, precision, and publication bias. The overall quality of outcomes in the studies will be assessed using GRADE, and results will be stratified as high (further research is very unlikely to change our confidence in the estimate of effect), moderate (further research is likely to have an important impact on our confidence in the estimate of effect and may change the estimate), low (further research is very likely to have an important impact on our confidence in the estimate of effect and is likely to change the estimate), or very low (very uncertain about the estimate of effect) [31]. GRADE will be conducted by 3 pairs of authors in which each pair will be independently performing the GRADE assessment for each citation with any disagreements resolved through discussion. If no consensus can be established, a third reviewer will be consulted.

### Data Synthesis

Tables will be constructed to describe the types of studies, populations, characteristics of the IPE intervention, professions involved in the intervention, comparator, and outcomes. Meta-analysis will be conducted if at least 2 studies reported the same outcome. The standardized mean difference will be the summary measure collected if applicable and measures of consistency will be explored via the *I*^2^ statistic. If the estimated *I*^2^ statistic is equal to or greater than 50% this would be interpreted as a large amount of heterogeneity [32]. Furthermore, due to the abundance of variability in IPE research concerning participants, interventions, comparators, and study designs, a random-effects model that inherently allows more heterogeneity will be used. If meta-analysis cannot be performed, the data will be synthesized through a narrative approach.

Subgroup analysis may be conducted if sufficient data are available from two or more studies to examine outcome variability and population affected by the outcome (patient-reported outcomes, clinician outcomes, and student outcomes) or exposure variability (setting, frequency, and duration; including the potential total number of exposure hours and whether hours came from patient-facing vs nonpatient-facing exposure experiences). Potential subgroup analyses may include examining the impact of an IPE intervention between different prelicensure rehabilitation science students. These subgroup analyses, if conducted, might inform the authors and readers further on the aspects of IPE that should be prioritized, and potentially some of the current best methods by which to measure the outcomes of interest from a collaborative and potentially patient experience perspective.

## Results

This project was initiated in November 2023, a search strategy was drafted on January 17, 2024, and trialed in a preliminary search in MEDLINE, before consulting with the Health Sciences Librarian at McMaster University on February 7, 2024. The search strategy was finalized on March 8, 2024, and this study was registered on PROSPERO on March 31, 2024 (CRD 42024506081). The final search across the 5 databases resulted in 10,692 citations for abstract and title screening. 756 citations were eligible for full-text review, which started in April 2024. From the eligible citations, 6 citations were considered for data extraction. Data extraction began in July 2024 and is ongoing. Data extraction will be finalized for knowledge synthesis by September 2024. This project is expected to be completed and finalized by October 2024 and a paper will be prepared for publication by November 2024.

## Discussion

To the authors’ knowledge, this is the first time a review focusing on IPE with prelicensure rehabilitation science students will be performed. Previous studies show the potential of IPE in improving collaboration, communication, and role clarity among interprofessional peers [8-10]. It is hypothesized that students in professional rehabilitation sciences programs will experience similar benefits. As there are limited synthesis reviews on this subgroup of students, our study will bring forward their perspectives on IPE effectiveness, possibly identifying the necessary IPE components for successful implementation. Rehabilitation professions play a crucial role in facilitating the prevention, treatment, and overall management of various health conditions [1,12], emphasizing the need for this study. Evaluating the effectiveness of these IPE interventions provides a greater understanding of students’ readiness and its associated improvement for IPC upon entering the workforce.

There are numerous IPE interventions described in the literature, and considerations for their implementation are usually generally described [3]. Comprehensive IPE activities need to consider each participating student’s role and responsibilities under relevant circumstances to allow for skill development in conflict resolution, skillful communication, and collaborative practice for improving patient care [3,6,10]. However, these contexts and nuances are rarely reported, especially from the perspective of rehabilitation science students. Thus, this review may also provide clarity on future learning environments, delivery methods, or implementation, which could be implemented to formalize rehabilitation science students’ awareness of their interprofessional identity and contributions to patient care [19].

Furthermore, the knowledge gained from this review may impact education for prelicensure rehabilitation science students by bringing forward the essential components and direction for IPE in their curriculums. There are several interprofessional competencies including role clarity, team-based collaborations, IPC, and values in interprofessional practices [7,33]. These competencies are interdependent and are necessary to facilitate high-quality patient care [5,6,33]. Thus, IPE delivery must be continuously reviewed for alignment with these outlined competencies to ensure prelicensure students are gaining the necessary experiences to facilitate their IPE readiness [7,19,34]. In addition, the promotion and implementation of IPE opportunities should be evaluated to ensure they address prelicensure rehabilitation science students’ needs as their roles differ from those of physicians and nurses [8,9,11]. By identifying the current IPE structure, delivery, and implementation, this information can identify rehabilitation science students’ knowledge gaps in core competencies, informing the future development of IPE in rehabilitation professional programs. Subsequently, this knowledge growth will supplement WHO’s mandate of using innovative approaches to teach health care students across the world to optimize IPE readiness in future generations of health care providers [4].

The proposed systematic review will have many strengths including the registration of the protocol prior to study commencement and the use of the Cochrane Handbook and PRISMA guidelines to optimize rigor and reporting transparency [13,31]. These outlined steps will facilitate the authors’ progress at all stages to mitigate errors, and risk of bias. This review will also include rehabilitation science students who participate in IPE interventions, where we used an international definition outlined by the WHO, a global health organization [12]. Since there are common outcome measures for interpreting perspectives on IPE interventions, there may be an opportunity for meta-analysis and determination of standardized outcome measures for IPE, an identified need in IPE research. This systematic review will include a variety of study designs and include studies published after 2016 to reflect the Institute of Medicine’s report on the impacts of IPE [7], capturing the most relevant and current evidence in IPE literature.

Potential limitations may include studies lacking long-term follow-up, varying populations included, and varying intervention methods. Furthermore, the included studies may not use the same outcome measures, reducing the possibility for further analyses via a meta-analysis. Our search is also limited by using the search term “healthcare” instead of “health” as the term “health” broadened our initial search by over 20,000 results in comparison to the term “healthcare.” With a high-level review of the search results using “health,” the scope of the papers was not directly applicable to this proposed systematic review, and the term “healthcare” was therefore used instead to optimize study feasibility and relevancy to professional health care programs.

## Appendix

Multimedia Appendix 1 Title and abstract tool.

Multimedia Appendix 2 Full text screening tool.

Multimedia Appendix 3 Inclusion and exclusion criteria.

Multimedia Appendix 4 MEDLINE search strategy.

Multimedia Appendix 5 Data extraction tool.

## Abbreviations

GRADE: Grading of Recommendations, Assessment, Development, and Evaluation
IPC: interprofessional collaboration
IPE: interprofessional education
MeSH: Medical Subject Headings
PICO: population, intervention, comparison, and outcome
PRISMA: Preferred Reporting Items for Systematic Reviews and Meta-Analysis
PRISMA-P: Preferred Reporting Items for Systematic Reviews and Meta-Analysis Protocols
RCT: randomized controlled trial
RoB: risk of bias
WHO: World Health Organization

## Data Availability

All data generated or analyzed during this study are included in this article and additional information may be available from the corresponding author upon reasonable request.
