# Supplementary material for: Effects of Interprofessional Education on Readiness for Interprofessional Learning in Rehabilitation Science Students From Professional Health Care Programs: Protocol for a Systematic Review
Source: JMIR Res Protoc. 2024 Nov 20;13:e60830. doi: 10.2196/60830 (PMC11618007; doi:10.2196/60830)
Supplement: Multimedia Appendix 1 [file resprot_v13i1e60830_app1.pdf]

Appendix 1: Title and Abstract Tool

1. Did the study take place in a healthcare setting?

   YES____ NO____ MAYBE____

2. Were multiple healthcare professions represented in the study?

   YES____ NO____ MAYBE____

3. Was there an interprofessional education intervention performed?

   YES____ NO____ MAYBE____

All studies that answered YES and MAYBE were included. Studies with at least one NO were not included.
