# Supplementary material for: Effects of Interprofessional Education on Readiness for Interprofessional Learning in Rehabilitation Science Students From Professional Health Care Programs: Protocol for a Systematic Review
Source: JMIR Res Protoc. 2024 Nov 20;13:e60830. doi: 10.2196/60830 (PMC11618007; doi:10.2196/60830)
Supplement: Multimedia Appendix 2 [file resprot_v13i1e60830_app2.pdf]

Appendix 2: Full Text Screening Tool

1. Is the full study available in either english or portuguese

   YES____ NO____

2. Does the study include outcomes for at least one of physiotherapists, occupational therapists, speech and language therapists and audiologists, orthotists and prosthetists, clinical psychologists, physical medicine and rehabilitation doctors, and rehabilitation nurses?

   YES____ NO____

3. Are the interventions performed in a pre-licensure setting?

   YES____ NO____

All studies that answered YES were included. Studies with at least one NO were not included.
