# Supplementary material for: Effects of Interprofessional Education on Readiness for Interprofessional Learning in Rehabilitation Science Students From Professional Health Care Programs: Protocol for a Systematic Review
Source: JMIR Res Protoc. 2024 Nov 20;13:e60830. doi: 10.2196/60830 (PMC11618007; doi:10.2196/60830)
Supplement: Multimedia Appendix 3 [file resprot_v13i1e60830_app3.pdf]

Appendix 3: Inclusion and Exclusion Criteria

CRITERION	INCLUSION	EXCLUSION
Population	Studies including pre-licensure students in entry-to-practice rehabilitation training programs. Rehab disciplines will include:<br>- Physiotherapists<br>- Occupational Therapists<br>- Speech Language Pathologists<br>- Audiologists<br>- Orthotists<br>- Prosthetists<br>- Clinical Psychologists<br>- Physical Medicine and Rehabilitation Doctors<br>- Rehabilitation Nurses	Studies that only include non-rehabilitation disciplines or only include licensed rehabilitation professionals already practicing in healthcare.
Intervention	Studies will be included if their IPE interventions include presenting two or more rehabilitation learners engaging in an active exchange of information and participation in	None

	activities aimed at improving communication and collaboration skills.	
Comparator	None	None
Outcomes of Interest	The primary outcome is readiness for interprofessional collaboration. This will be measured using outcome measures including but not limited to:<br>- Readiness for Interprofessional Learning Scale (RIPLS)<br>- Student Perceptions of Interprofessional Clinical Education Revised (SPICE-R)<br>- Interdisciplinary Education Perception Scale (IEPS)	Studies that did not address outcome measures related to readiness towards interprofessional collaboration (primary outcome) or secondary outcomes as mentioned to the left.

	- Entry Level Interprofessional Questionnaire (ELIQ)<br>- Brief Attitudes Survey for Interprofessional Collaborative Learning (BASIC-L)<br>The secondary outcomes are students' perception of interprofessional collaboration, students' appreciation for the role and scope of complimentary rehabilitation disciplines, closed-loop communication, confidence levels, practice efficiency, and team dynamics. These will be measured using outcome measures including but not limited to:<br>- Anesthetists' No technical skills (ANTS)<br>- Appropriate checklists/questionnaires	

Study Design	Any peer-reviewed and published study design including but not limited to:<br>- Randomized control trials<br>- Cohort studies<br>- Case control studies	Narrative reviews, scoping reviews, systematic reviews, Abstracts, dissertations, theses, editorials, conference proceedings, magazines, news, and any other non-peer-reviewed documents.
Language of Publication	Studies available in English and Portuguese.	Studies only available in languages other than English or Portuguese.
Date Limits	All available articles from 2016-present will be included from the following but not limited to databases:<br>- Medline<br>- CINAHL<br>- AMED<br>- Embase<br>- ERIC	Studies published prior to the year 2000.
