# Supplementary material for: Effects of Interprofessional Education on Readiness for Interprofessional Learning in Rehabilitation Science Students From Professional Health Care Programs: Protocol for a Systematic Review
Source: JMIR Res Protoc. 2024 Nov 20;13:e60830. doi: 10.2196/60830 (PMC11618007; doi:10.2196/60830)
Supplement: Multimedia Appendix 4 [file resprot_v13i1e60830_app4.pdf]

## Appendix 4: Medline Search Strategy

Search Strategy Limits: Publication Year: 2000-Current

Language: English and Portuguese

Ovid MEDLINE(R) ALL <1946 to February 28, 2024 at 10:00pm EST>

1 interprofession*.mp. 64625

2 interdiscipli*.mp. 68612

3 interdepart*.mp. 2123

4 inter occupation*.mp. 12

5 multidisciplin*.mp. 124773

6 multiprofession*.mp. 1986

7 education*.mp. 1210564

8 allied healthcare.mp. 466

9 healthcare team.mp. 2969

10 allied healthcare occupations.mp. or exp Allied Health Occupations/ 53822

11 allied healthcare person.mp. or exp Allied Health Personnel/ 54838

12 physiotherap*.mp. 36085

13 physical therapy.mp. 60903

14 exp Physical Therapists/ or physical ther*.mp. 67138

15 exp Audiology/ or exp Audiologists/ or audiolog*.mp. 14258

16 exp Rehabilitation/ or rehab*.mp. 630098

17 occupational therap*.mp. or exp Occupational Therapy/ 24574

18 occupational therapist.mp. or exp Occupational Therapists/ 2476

19 exp Speech-Language Pathology/ or speech language patholo*.mp. 6662

20 speech language pathologist.mp. 884

21 orthotist*.mp. 272

22 prosthetist*.mp. 496

23 exp Psychology, Clinical/ or clinical psychologist*.mp. 4962

24 physical medicine doctors.mp. 1

25 exp "Physical and Rehabilitation Medicine"/ or physical medicine rehabilitation.mp. 48399

26 exp Physiatrists/ or physiatrist*.mp. 1534

27 Physiatr*.mp. 2277

28 exp Rehabilitation Nursing/ or rehabilitation nurs*.mp. 2279

29 recreational therapy.mp. or exp Recreation Therapy/ 215

30 recreational therapist*.mp. 28

31 students.mp. or exp Students/ or exp Students, Health Occupations/ 374236

32 learne*.mp. 114687

33 exp Learning/ or learners.mp. or exp Curriculum/ 540230

34 health sci* learn*.mp. 14

35 exp Universities/ or universit*.mp. 519709

36 1 or 2 or 3 or 4 or 5 or 6 248440

37 7 and 36 48024

38 8 or 9 or 10 or 11 or 12 or 13 or 14 or 15 or 16 or 17 or 18 or 19 or 20 or 21 or 22 or 23 or 24 or 25 or 26 or 27 or 28 or 29 or 30 768895

39 31 or 32 or 33 or 34 or 35 1338417

40 37 and 38 and 39 2292

| | | |
|---|---|---|
| 41 | limit 40 to yr="2000 -Current" | 2005 |
| 42 | limit 41 to (english or portuguese) | 1919 |
