# Supplementary material for: Effects of Interprofessional Education on Readiness for Interprofessional Learning in Rehabilitation Science Students From Professional Health Care Programs: Protocol for a Systematic Review
Source: JMIR Res Protoc. 2024 Nov 20;13:e60830. doi: 10.2196/60830 (PMC11618007; doi:10.2196/60830)
Supplement: Multimedia Appendix 5 [file resprot_v13i1e60830_app5.pdf]

Appendix 5: Data Extraction Tool

Study Characteristics:

Study Background Info				Total Sample Characteristics	
Study Number	Author, Year	Country	Study Design	Sample Size	Age (SD), sex (%)

Intervention Characteristics:

Background Info			Experimental Group			
Study Number	Author, Year, Country	Length of Study	Setting	Intervention Components	Frequency	Duration of Session

Control				Data Collection	
Setting	Components	Frequency	Duration of Session	Timing of Data Collection	Outcome Measure Used

Primary Results

Study Details		Outcome Measure		Results
Study Number	Author, Year, Country	Outcome Measure	Outcome Measure Subscale	Baseline Mean (experimental)

Group Results					
Baseline Mean (control)	Baseline SD (control)	Post-intervention mean (experimental)	post-intervention SD (experimental)	Post-intervention Mean (control)	Post-intervention SD (control)

Comparative Results				
Group difference mean	Group difference SD	Group difference p-value	Effect Size	Summary of Result

Secondary Results

JMIR ms#60830

Study Details		Outcome Measure		Results
Study Number	Author, Year, Country	Outcome Measure	Outcome Measure Subscale	Baseline Mean (experimental)

Group Results					
Baseline Mean (control)	Baseline SD (control)	Post-intervention mean (experimental)	post-intervention SD (experimental)	Post-intervention Mean (control)	Post-intervention SD (control)

Comparative Results				
Group difference mean	Group difference SD	Group difference p-value	Effect Size	Summary of Result
